# Genome Sequence of *Ureaplasma diversum* Strain ATCC 49782

**DOI:** 10.1128/genomeA.00314-15

**Published:** 2015-04-16

**Authors:** Lucas M. Marques, Ana M. S. Guimarães, Hellen B. Martins, Izadora S. Rezende, Maysa S. Barbosa, Guilherme B. Campos, Naíla C. do Nascimento, Andrea P. dos Santos, Aline T. Amorim, Verena M. Santos, Joanne B. Messick, Jorge Timenetsky

**Affiliations:** a Department of Microbiology, Institute of Biomedical Science, University of São Paulo, São Paulo, Brazil; b Multidisciplinary Institute of Health, Universidade Federal da Bahia, Vitória da Conquista, Brazil; c Department of Animal Health and Preventive Veterinary Medicine, College of Veterinary Medicine and Animal Science, University of São Paulo, São Paulo, Brazil; d Department of Comparative Pathobiology, Purdue University, West Lafayette, Indiana, USA

## Abstract

Here, we report the complete genome sequence of *Ureaplasma diversum* strain ATCC 49782. This species is of bovine origin, having an association with reproductive disorders in cattle, including placentitis, fetal alveolitis, abortion, and birth of weak calves. It has a small circular chromosome of 975,425 bp.

## GENOME ANNOUNCEMENT

*Ureaplasma diversum* is a bovine-origin ureaplasma isolated in 1969. Initially, it was defined as a nonpathogenic species, but it recently has been shown to cause damage in bovine organs and cells (1–5). This ureaplasma has been associated with female infertility, placentitis, fetal alveolitis, and abortion or birth of weak calves (3–5). In bulls, it is an important pathogen of the genital tract and may cause low sperm motility, seminal vesiculitis, and epididymitis (1, 6). Nevertheless, the mechanisms by which this organism exerts its virulence and pathogenicity in cattle are mostly unknown (1, 7–9).

*U. diversum* ATCC 49782 was first cultured in 2 ml of *Ureaplasma* medium (UB) at 37°C, followed by propagation in 3,000 ml of the same broth. At the logarithmic growth phase (based on colorimetric changes), the culture was centrifuged at 20,600 × *g* for 30 min at 25°C. The DNA was extracted using a PureLink genomic DNA minikit (Life Technologies, Brazil). The whole genome was sequenced from a paired-end library using Illumina HiSeq 2000 (Illumina, Inc., San Diego, CA) at the Purdue University Genomics Core Facility. Average reads of about 100 bases were assembled using ABySS 1.2.7. After assembly, resulting in 4,552× genome coverage, eight remaining gaps were closed using conventional PCR, followed by Sanger sequencing in both directions. First-pass annotation was achieved using the National Center for Biotechnology Information (NCBI) Prokaryotic Genome Annotation Pipeline (PGAP).

The complete genome of *U. diversum* strain ATCC 49782 is composed of 975,425 bp in a single circular chromosome, with a G+C content of 28.1%. It appears to use the opal stop codon (UGA) for tryptophan. A total of 646 coding DNA sequences (CDSs), 6 rRNAs, and 22 tRNA genes were predicted and annotated. Three hundred seventy-four CDSs (57.9%) have putative functional identities; however, 272 CDSs (42.1%) are hypothetical proteins.

Comparing the data obtained for *U. diversum* ATCC 49782 with those for 14 ureaplasma serovars of human origin (10 serovars of *Ureaplasma urealyticum* and 4 serovars of *Ureaplasma parvum*) (10), a larger genome size was observed (*U. diversum*, 0.97 Mbp; *U. urealyticum*, 0.84 to 0.95 Mbp; *U. parvum*, 0.75 to 0.78 Mbp), with a slightly higher G+C content (*U. diversum*, 28.1%; *U. urealyticum*, 25 to 27%; *U. parvum*, 25%), number of CDSs (*U. diversum*, 646; *U. urealyticum*, average of 608; *U. parvum*, average of 608), and number of hypothetical CDSs (*U. diversum*, 237; *U. urealyticum*, average of 230; *U. parvum*, average of 201). These genetic differences may reflect for the host specificity of *U. diversum*. However, a detailed assessment of the genes needs to be performed.

The sequence described here represents a valuable new resource for the study of *U. diversum* on a genetic basis. Further, extensive analyses of these data may help us to better understand the host specificity of this mollicute and what defines its pathogenicity in bovines. Moreover, it will also aid the development of new strategies for the treatment, prevention, and control of ureaplasmal infections.

### Nucleotide sequence accession number. 

The genome sequence of *U. diversum* strain ATCC 49782 has been deposited in GenBank under the accession no. CP009770.
